# The Role of Food Structure on Fatty Acid Bioaccessibility: A Decade of TIM‐1 Simulated Digestion Studies in Review

**DOI:** 10.1002/mnfr.70434

**Published:** 2026-03-15

**Authors:** Michael A. Rogers, Amanda J. Wright

**Affiliations:** ^1^ Department of Food Science University of Guelph Guelph ON Canada; ^2^ Human Health and Nutritional Science University of Guelph Guelph ON Canada

**Keywords:** bioaccessibility, emulsifiers, food structure, lipemic response, lipid digestion, sn‐2 monoglycerides, ultra‐processed foods

## Abstract

Food structure has emerged as a critical concept with wide‐ranging implications for nutrition and health. Studies with in vitro digestion models are enabling a better fundamental understanding of the structure–function relationships that define how food matrix properties change and influence nutrient release and transit during gastrointestinal digestion. Dietary lipids are of particular relevance due to their high energy density and associations with cardiometabolic risk, including postprandial rises in blood lipids. Studies using the dynamic TIM‐1 digestion model enable investigations of fatty acid bioaccessibility, a precursor to lipid absorption, across a range of food products. This review presents the results of several investigations into food structure, with TIM‐1 focusing on lipid digestion and instances where the findings correlate with corresponding human studies. Collectively, a better understanding of how food structure influences postprandial lipemia supports the development of food products tailored to benefit health.

## Graphical abstract

Lipid and food structure determine when and how macro‐ and micronutrients become bioaccessible. This review compares lipid bioaccessibility from engineered emulsions, breast milk, infant formula, and meat and meat mimics, which couples the effect of cooking temperature on lipid bioaccessibility in meat and eggs.

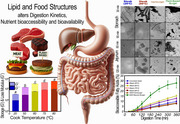

**Abbreviations**
2‐MAG: 2‐monoglyceride
ACW: antral contraction waves
APO: apolipoprotein
AUC: area under the curve
BLG: β‐lactoglobulin
BM: breastmilk
CIE: chemical interesterification
C_max_
: maximum bioaccessible fraction
CR: maximum crystallinity emulsion
cvd: cardiovascular disease
D(4,3): volume‐weighted mean
DAG: diglyceride
DOD: degree of doneness
DSC: differential scanning calorimetry
EIE: enzymatic interesterification
FA: fatty acid
FTIR: Fourier‐transform infrared spectroscopy
G`: storage modulus
G``: loss modulus
GI: glycemic index
GLP‐1: glucagon‐like peptide‐1
HCL: hydrochloric acid
HDL: high‐density lipoprotein
HLB: hydrophilic–lipophilic balance
HMF: human milk fat
IDL: intermediate‐density lipoprotein
IF: infant formula
LCFA: long‐chain fatty acids
LDL: low‐density lipoprotein
LI: lipemic index
LU: undercooled acid‐unstable emulsion
LS: undercooled acid‐stable emulsion
LysoPC: lysophosphatidylcholine
MCT: medium‐chain triglycerides
MFGM: milk fat globular membrane
MUFA: monounsaturated fatty acid
NIE: non‐interesterified
O/W: oil‐in‐water
PO: palm olein
PPY: polypeptide Y
PUFA: polyunsaturated
PS: palm stearin fatty acid
PSD: particle size distributions
PS‐LE: undercooled PS emulsion
PS‐SE: crystalline PS emulsion
PYY: peptide YY
SCFA: short‐chain fatty acids
SFA: saturated fatty acids
SFL: solid fat content
SLS: sodium lauryl sulphate
Span 20: sorbitan monolaurate
Span 60: sorbitan monostearate
Span 80: sorbitan monooleate
SU: acid‐unstable, partially crystalline emulsion
SS: acid‐stable, partially crystalline emulsions
SSF: sodium stearyl fumarate
SSL: sodium stearoyl lactylate
TAG: triglyceride
TIM‐1: TNO intestinal model
UC: undercooled liquid emulsion
VLDL: very low‐density lipoprotein

## 1 Introduction

Postprandial triglycerides (TAGs) are an independent cardiovascular disease (CVD) risk factor, potentially more relevant than fasting plasma TAGs for early CVD risk detection [1, 2]; while epidemiological studies show hypertriglyceridemia is a risk factor for atherosclerosis and cerebrovascular disease [3, 4]. Fasting plasma TAGs reflect the chronic effects of diets and metabolic activity but not the postprandial elevations of circulating TAGs following a meal [5]. After a high‐fat meal, TAG elevations vary significantly among individuals, depending on visceral adiposity, insulin resistance, and the frequency of aerobic exercise [6]. However, a high‐fat meal typically increases circulating TAG levels by ∼50% relative to fasting levels, making postprandial TAG elevations a relevant biomarker for health [7]. Given the implications of postprandial lipemia, developing a lipemic index (LI) akin to the widely used glycemic index (GI) to measure the effect of carbohydrates on serum glucose levels is a pressing need in nutrition and health [4, 8]. The postprandial state, or the period after a meal when the body digests and absorbs nutrients, is especially relevant to health and imperative to consider, as individuals are non‐fasting for ∼18 h daily [3]. Meals with a low glycemic response may still contain significant lipids and an exaggerated lipemic response, helping rationalize the development of a standardized LI that characterizes the lipemic response of foods or meals [1, 9]. A helpful LI should account for the postprandial increase in serum TAGs after a test meal, compared to a reference meal containing a fixed load of saturated, monounsaturated, and polyunsaturated fats in known proportions [4]. A single meal with ^13^C‐labelled fatty acids (FA) showed that FAs absorption rates differ (oleate > palmitate > linoleate) [10].

In addition to meal composition, including the total amount of dietary fat, apolar lipids, fatty acids, and TAG composition [11, 12], the physical structure of foods and lipids within them changes along the gastrointestinal tract, impacting postprandial lipemia [5, 13]. Before absorption is possible, the food structure must erode sufficiently to expose the TAGs confined within the food matrix to digestive fluids. The apolar nature of TAGs limits their aqueous solubility, meaning that dietary TAGs need to be dispersed and broken down within the gastrointestinal tract before absorption. Specifically, ingested TAGs hydrolyze into FAs and sn‐2‐monoglycerides (2‐MAGs), which are then absorbed through the intestinal villi and assimilated back into TAGs, where long‐chain fatty acids are incorporated into chylomicrons with apolipoprotein B48 (ApoB48) and transported through the lymphatic circulation before entering circulation. Once chylomicron remnants reach the liver, they get repackaged into very low‐density lipoprotein (VLDL), with ApoB100 contributing to hepatic TAGs during postprandial lipoproteinemia [14, 15].

**FIGURE 1:**
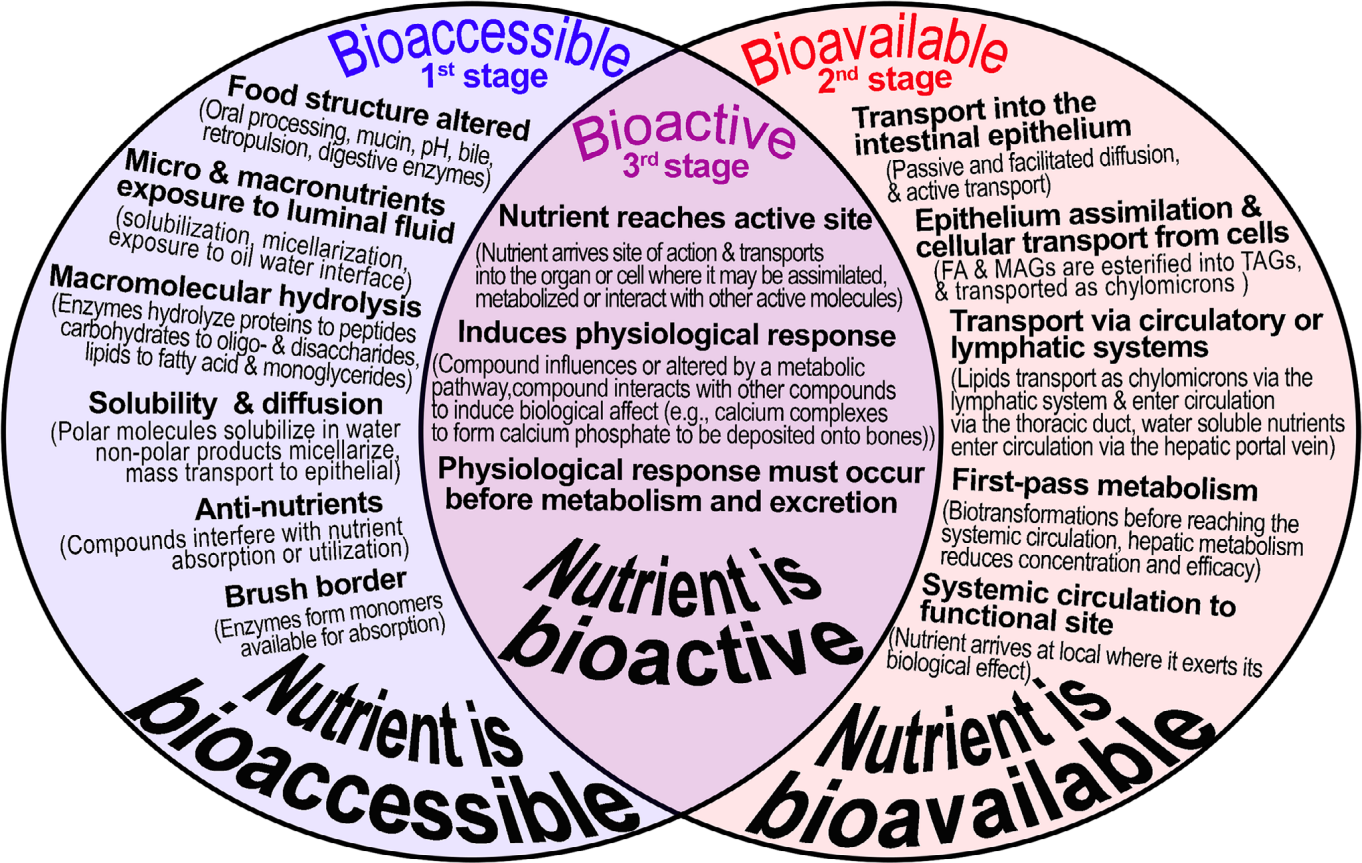
Factors influencing bioaccessibility, bioavailability, and bioactivity.

Bioaccessibility as a potential rate‐limiting step impacting lipemic response: The benefit of simulating pre‐absorptive aspects of digestion. Bioaccessibility refers to the nutrients released from the food matrix into the luminal fluid, available for absorption during digestion (Figure 1). In contrast, bioavailability refers to the nutrients that are absorbed and available to perform their intended physiological functions [16, 17, 18]. Two foods with equivalent chemical compositions may differ in healthfulness and nutritional outcomes due to the “food matrix effect” [19]. The “food matrix effect” alters bioaccessibility, a precursor to bioavailability, which depends on pre‐adsorptive digestive conditions in which food transforms sequentially from the swallowed bolus to chyme and then to digestate, thereby facilitating the exposure of macronutrients to digestive enzymes. The breakdown of physical structures during digestion is a precursor to bioaccessibility as the structure must erode before digestive enzymes in luminal fluids access macronutrients. Factors may delay or expedite lipid digestion and absorption, thus affecting the duration and intensity of postprandial lipemia. Therefore, standardized postprandial lipemia markers must account for the food structure that incorporates lipids. These parameters include lipid composition, quantity, and physical state [5]. Engineering food structure indirectly affects bioavailability, as tightly regulated homeostatic pathways govern post‐absorptive processes. These processes include assimilation of FA and sn‐2‐MAGs into TAGs, lymphatic and circulatory transport, first‐pass metabolism, and utilization at active sites. In other words, food structure modulates the exposure of nutrients to luminal fluids, thereby altering the rates of hydrolysis, solubilization, and diffusion to the intestinal epithelium. It may act as the rate‐limiting step in nutrient bioavailability. Since bioaccessibility precedes bioavailability, the rate of nutrient release into the luminal fluid directly influences bioavailability [20].

Beyond composition, the macroscale rheological properties imparted by food structure (e.g., hardness, brittleness, elasticity), lipid physical state (i.e., liquid or solid), interfacial characteristics of emulsions (e.g., size and distribution of the dispersed lipid droplet or colloidal fat crystals), and the presence of emulsifier (e.g., zeta‐potential, steric repulsion, and interfacial tension) [21, 22] alter lipid digestion kinetics [23, 24, 25]. Ingested foods are successively altered along the alimentary tract, continuously reducing the hierarchical food and lipid structures and particle size until digestive enzymes access individual macronutrients, which hydrolyze into monomers (e.g., glucose, FA, 2‐MAG, amino acids) that become bioaccessible and available for absorption [26]. FA and 2‐MAGs incorporate into micelles in the luminal fluid, becoming bioaccessible and available for absorption; the onset of bioavailability concurs with mixed micelle transport into intestinal epithelium cells, where assimilation reesterifies the long‐chain FA (LCFA) and MAGs forming TAGs, which enter the lymphatic system as chylomicrons (e.g., LCFA) before reaching the thoracic duct and entering circulation. As lipids shuttle through the exogenous pathway as mixed micelles of bile and dietary lipids, lipoprotein lipase hydrolyzes fatty acids from triacylglycerols (TAGs) for utilization by adipose tissue. Chylomicron remnants are taken up by the liver and released as VLDL, initiating the endogenous pathway for circulating lipids as bile micelles. Lipoprotein lipase hydrolyzes fatty acids from circulating VLDL for use by muscle and adipose tissues, converting these particles to intermediate‐density lipoprotein (IDL) and eventually the cholesterol‐rich low‐density lipoprotein (LDL), which interacts with high‐density lipoprotein (HDL) to return cholesterol to the liver, that is, reverse cholesterol transport.

### 1.1 The Dynamic TIM‐1 Simulated Gastrointestinal Tract

**FIGURE 2:**
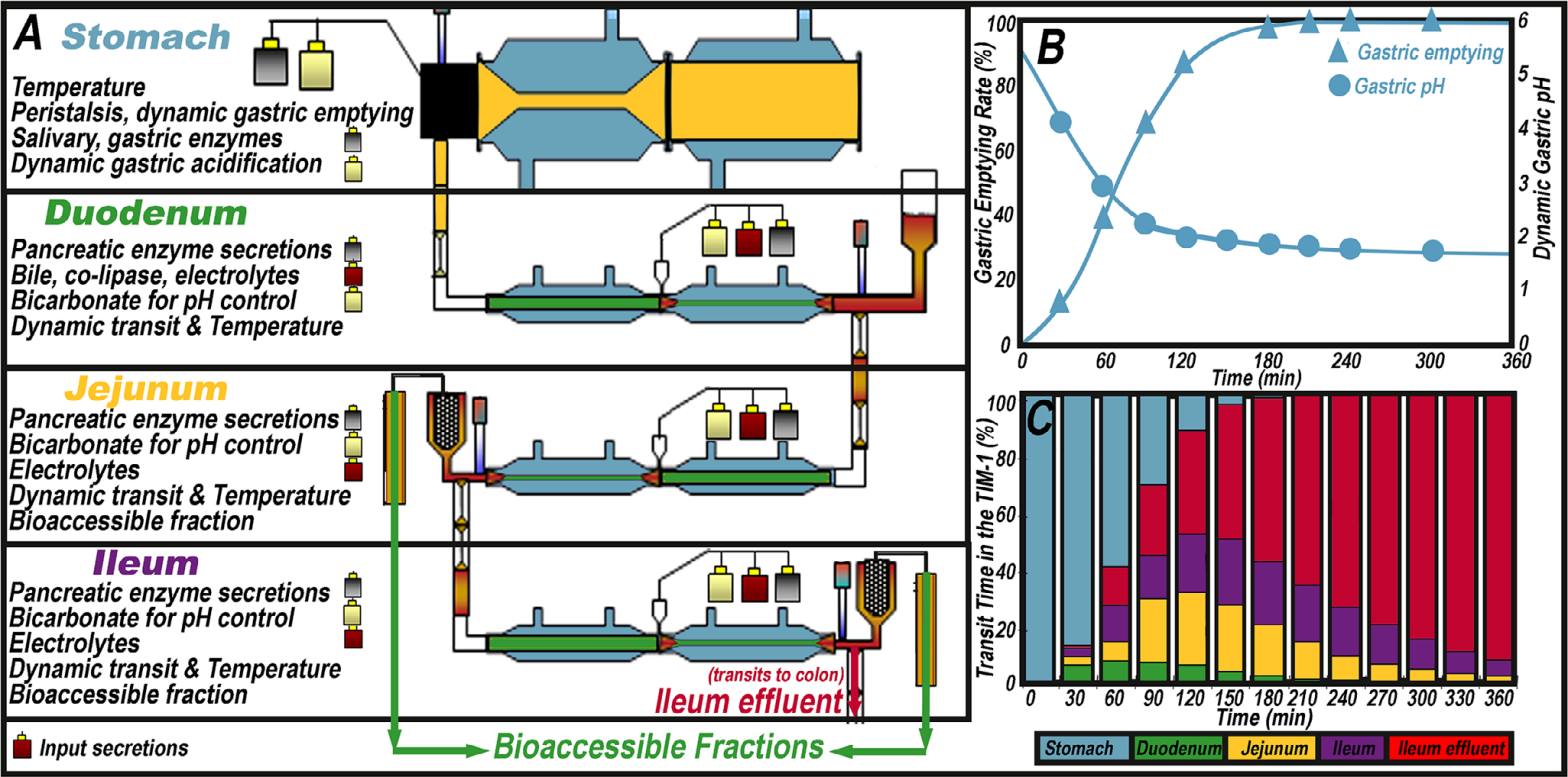
Dynamically controlled parameters in the TIM‐1 compartments (A), including the gastric acidification and emptying rates (B) and transit time through each TIM‐1 compartment (C).

Many systems simulate conditions in the gastrointestinal tract, ranging from simple single‐compartment systems to more complex models. Most in vitro models do not simulate the entire gastrointestinal tract; however, the TNO intestinal model (TIM‐1) systems cover most digestive elements up to absorption, except for oral processing and the large intestine, which TIM‐2 simulates [27]. The TIM‐1 is a multi‐compartmental system that accurately simulates key dynamic physico‐chemical conditions in the human gastrointestinal tract across various postprandial states (e.g., fed or fasted), ages (infant or healthy adult), and health statuses [28]. Validation studies of nutrients and pharmaceuticals widely use luminal concentrations and bioaccessibility to provide high predictive value for in vivo studies [28]. The luminal digestive conditions of the stomach, duodenum, jejunum, and ileum are highly reproducible, including pH and gastric, biliary, and pancreatic secretions (Figure 2A) [29]. The original TIM‐1 protocol simulates conditions of a typical healthy adult. Altered TIM‐1 conditions include disease models associated with severe pancreatic insufficiency, characterized by reduced bile and pancreatic lipase concentrations. Under these modified conditions, ciprofloxacin bioaccessibility in a lipid formulation differed [30, 31].

The TIM‐1 has four serially connected compartments, consisting of two glass units and a flexible inner silicone membrane. Circulating water between the glass jacket and silicone membrane under alternating pressure maintains the temperature at 37.0 ± 1.0°C and induces peristaltic‐like contractions [32]. Peristaltic valves dynamically control gastric emptying rate, typically with a half‐time of 80 min (Figure 2B), and intestinal transit time (Figure 2C). Gastric acidification with 1 M hydrochloric acid follows a pre‐set dynamic pH profile, acidifying the chyme from its initial pH to ∼1.7. The duodenal, jejunal, and ileal compartments maintain pH at 6.5, 6.8, and 7.2, respectively, using 1 M sodium bicarbonate. Here, we define the TIM‐1 bioaccessible lipid fraction as the proportion of FA that passively moves through the semipermeable capillary membranes (0.05 µm pore size or 6–12 kDa) attached to the jejunum and ileum compartments. Since the filters permit only passively transported micellerized lipids, the permeate FA concentration is in equilibrium with the lumen concentration; therefore, it is a closer approximation of bioaccessibility as opposed to bioavailability, which involves active transport and first‐pass metabolism in the liver. The benefits of the TIM‐1 include identical luminal conditions between replicates and variables, enabling the comparison of bioaccessibility between different formulations or food structures [28, 33].

Validation studies correlate in vitro bioaccessibility of TIM‐1 with in vivo data for macronutrients (e.g., lipids, carbohydrates, protein), micronutrients (e.g., calcium, folate, iron, lycopene, polyphenols), and pharmaceuticals (e.g., Favipiravir, Bisoprolol, metoprolol succinate) [17, 33, 34, 35, 36, 37]. Evaluating bioaccessibility between formulations during TIM‐1 digestion after 3 h, compared to in vivo results, correctly predicted the rank order for the area under the curve (AUC) (84%) and maximum bioaccessible fraction (*C*_max_) (79%) [34] The tightly regulated, reproducible gastrointestinal environment simulated by the TIM‐1 lacks the individual variability observed in human volunteers in clinical cohort studies [29]. An advantage is that it can isolate the effects of food composition and structure on lipid bioaccessibility. It does not account for variations observed in human clinical trials, including body mass, anatomy, or digestive motility [29]. The TIM‐1 describes a narrower physiological design space than a clinical study, given the tightly controlled, reproducible simulated gastrointestinal environment, which is ideal for studying the role of food structure on nutrient release [29].

### 1.2 Pre‐Duodenal Lipid Digestion Phase

**FIGURE 3:**
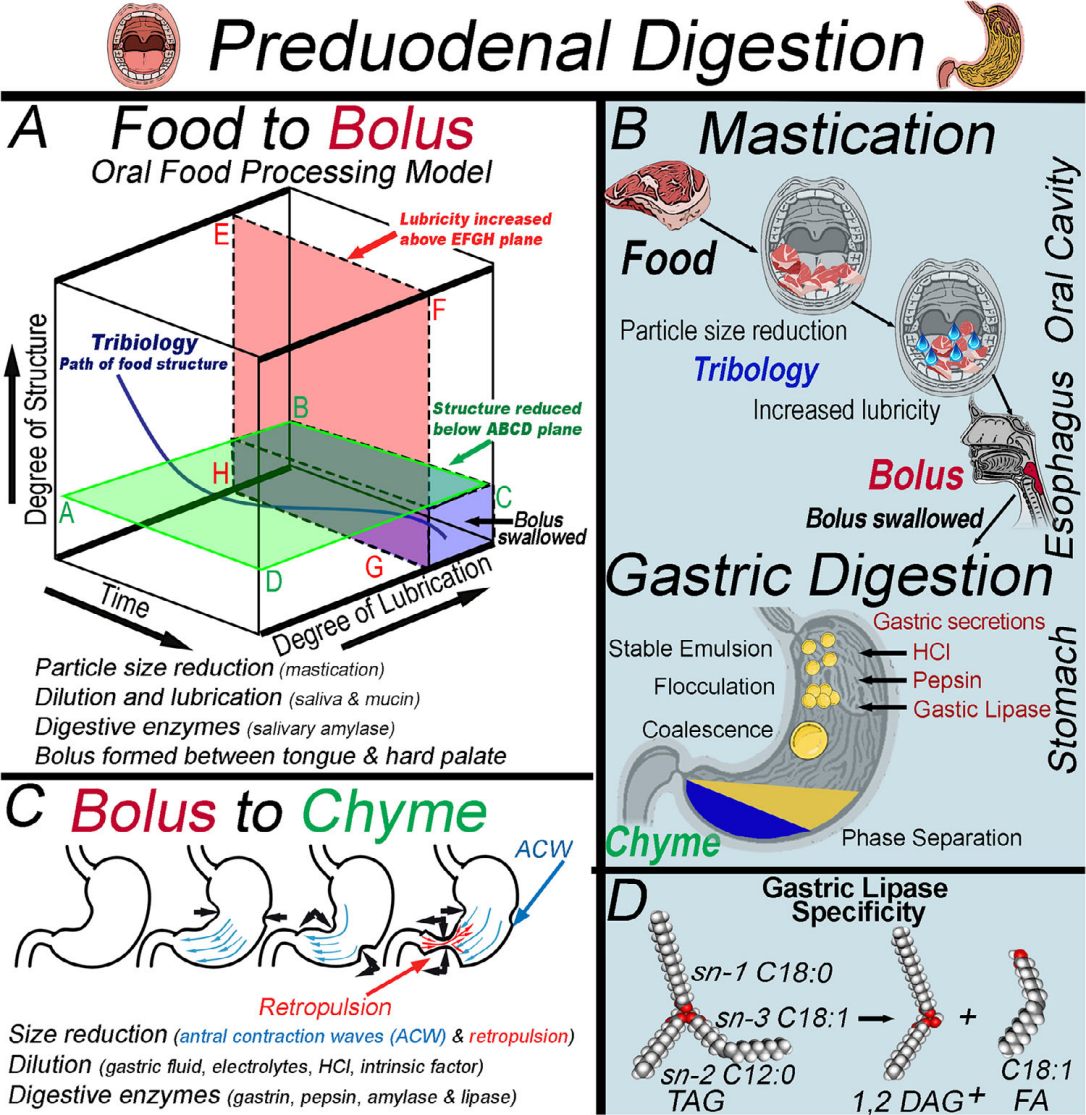
The oral food processing model (A) involves particle size reduction and lubrication to transform the food into a bolus before swallowing (B). Afterward, the bolus converts to chyme via antral contraction waves (C), which expose TAGs to acidic lipases that cleave the sn‐3 FA (D).

Gastrointestinal digestion involves a pre‐duodenal phase, encompassing the oral and gastric compartments (Figure 3) [38]. Oral processing models incorporate chewing to mechanically reduce particle size below a minimum threshold located below the ABCD plane. At the same time, salivary secretions dilute and lubricate above the EFGH plane (Figure 3A) [39], forming a bolus through manipulation of the tongue and hard palate before swallowing [40]. In the oral cavity, solid or semi‐solid foods require three phases of swallowing, with a minimum of two swallows preceded and followed by chewing cycles [41]. Saliva has a pH of 5–7 and is predominantly water with salts, immunoglobulins, proteins, and mucin (characterized by a large molecular mass, polydispersity, and a highly glycosylated glycoprotein that exhibits colloidal behavior (2–50 MDa)) [42, 43]. Mucin contains various functional domains, each capable of different noncovalent interactions (e.g., hydrophobic, electrostatic, and hydrogen‐bonding) that interact with other polymers in complex ways [42]. The mucin and salts in saliva are relevant to digestion, as mucin complexes with interfacial surfactants and proteins at the oil–water interface of emulsions, reducing interfacial tension and causing droplet bridging and depletion flocculation (Figure 3B) [44]. Additionally, salts reduce electrostatic repulsion between emulsions before they reach the stomach [45]. Saliva contains salivary α‐amylase, which initiates the hydrolysis of carbohydrates and is encoded by the AMY‐1 gene. This gene shows variation in concentration across populations, and gene duplication contributes to increased salivary amylase levels [46, 47]. Elevated salivary α‐amylase activity is associated with a reduced risk of obesity [48] and an increased prevalence of dental caries [46]. Oral lipolysis activity does not require bile salts or colipase. It remains active in the gastric compartment and is stereospecific for the sn‐3 ester, liberating one FA from the TAG and forming a sn‐1,2 diglyceride (DAG) [38]. Oral lipolysis is more specific for medium‐chain triglycerides (MCT), which are hydrolyzed 5–8‐fold faster than long‐chain triglycerides. In infants, this pathway accounts for a larger fraction of lipolysis due to limited pancreatic lipase activity, whereas in adults, its quantitative role is relatively minor but remains physiologically relevant [38].

Upon swallowing, the bolus transits through the esophagus to the stomach, where gastric juice containing HCl, salts, mucin, and gastric lipase acidifies the contents to a pH of 1–3. The bolus mixes with gastric fluid during antral contraction waves (ACWs), which originate from multiple sites in the upper corpus at a rate of 3 cycles per minute and propagate toward the pylorus (Figure 3C). As ACWs move distally, reaching the antrum, their amplitude and velocity increase until the pre‐pyloric stomach contracts, causing fluids to flow rapidly toward the narrowed pylorus and, subsequently, retropulsion, mixing the bolus and gastric fluid until they reach the consistency of chyme. The pylorus regulates the passage of chyme to the proximal end of the duodenum, controls gastric emptying rate, and determines the particle size of the chyme while preventing backflow from the duodenum to the stomach [49]. Gastric emptying rate depends on the disintegration of a food structure, as the pylorus limits the passage of particles greater than 1–2 mm into the duodenum, thereby altering nutrient absorption [49]. In healthy adults, gastric lipase accounts for approximately 10%–30% of TAG hydrolysis, specifically cleaving the sn‐3 ester bond, and preferentially releases SCFA and MCFA (Figure 3D) [38, 50, 51, 52]. During gastric TAG hydrolysis, concurrent peristaltic contractions induce mechanical and shear forces coupled with the highly acidic gastric fluid, causing a reduction in emulsion stability when surface proteins reach their isoelectric point, triggering flocculation [53]. The limited TAG hydrolysis observed during pre‐duodenal digestion does not negate the importance of this stage for subsequent hydrolysis, as the products promote TAG emulsification, alter lipid droplet interfacial properties, and facilitate colipase access to the emulsified lipid [54].

### 1.3 Intestinal Lipid Digestion Phase

**FIGURE 4:**
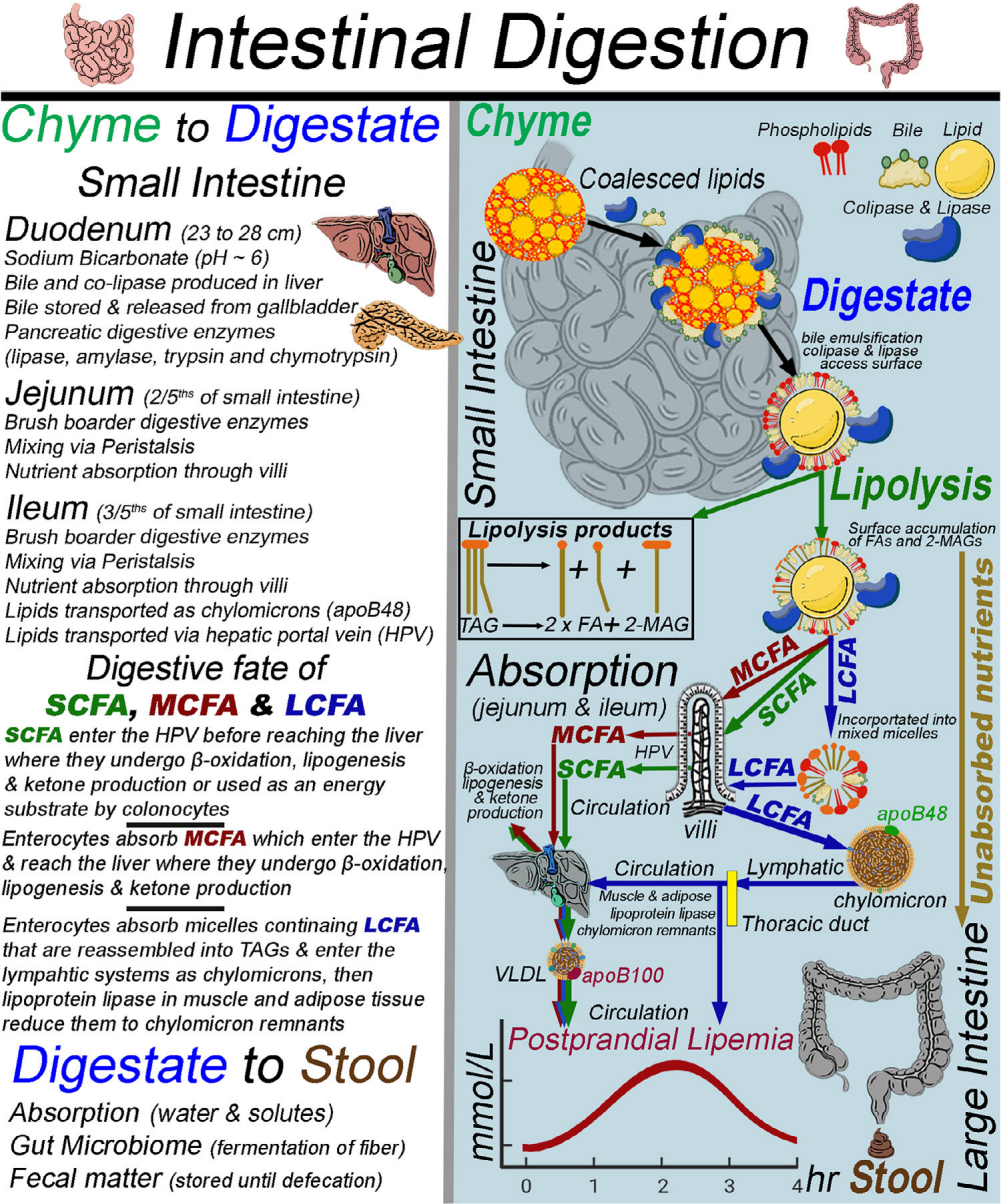
Biological fate of long‐, medium‐, and short‐chain fatty acids in the small and large intestine and their role in increasing postprandial lipemia.

Once the chyme passes through the pylorus, it enters the duodenum, where it mixes with digestive fluids and is neutralized to a pH of ∼ 6. Digestive fluids also contain bile, colipase, and pancreatic enzymes, including lipase, amylase, and proteases (trypsin, chymotrypsin, and carboxypeptidase). These molecules initiate the intestinal phase of digestion (Figure 4) [47]. Bile aids in emulsifying TAGs into smaller droplets, thereby increasing the surface area for colipase and lipase to access and, within the aqueous phase, contributes to the formation of mixed micelles [52]. TAGs are then preferentially cleaved at sn‐1 and sn‐3, resulting in an intermediate 1,2‐DAG before complete hydrolysis to a 2‐MAG and two FAs [52]. Other pancreatic lipases include pancreatic lipase‐related protein‐1, an inactive lipase due to mutations, pancreatic lipase‐related protein‐2, phospholipase A2, and carboxyl ester lipase, which are more relevant to neonatal digestion [52]. Enterocytes absorb mixed micelles containing FA, 2‐MAGs, cholesterol, phospholipids, and bile. Inside enterocytes, FAs are re‐esterified onto 2‐MAGs, forming TAGs, which are packaged into chylomicrons with Apolipoprotein B48 (ApoB48) and secreted into the lymphatic system, then reach circulation via the thoracic duct. Once circulating TAGs reach the capillaries of adipose and muscle tissues, endothelial lipoprotein lipase (LPL) hydrolyzes them, releasing free FAs, which are taken up by nearby tissues, and glycerol, which returns to the liver. When chylomicron remnants reach the liver, their contents are repurposed to synthesize VLDL. Long‐chain fatty acids (LCFA) (C14–22) are transported in chylomicrons. In contrast, short‐chain FAs (C2–C4) and medium‐chain FAs (MCFA) (C6–C12) are absorbed directly into the hepatic portal vein, circulating as free FAs complexed to plasma albumin; when they reach the liver, these FAs can undergo β‐oxidation, be used for lipogenesis, or be converted to ketone bodies, altering energy metabolism [16, 55, 56]. SCFAs, uncommon in dietary TAGs, are common metabolites formed by the gut microbiome during the anaerobic fermentation of indigestible polysaccharides and serve as an energy substrate by either colonocytes or hepatocytes [57, 58].

### 1.4 Fatty Acid Bioaccessibility in Structured Lipids

#### 1.4.1 Effect of Emulsifier Structure in Oil‐in‐Water Emulsions

**FIGURE 5:**
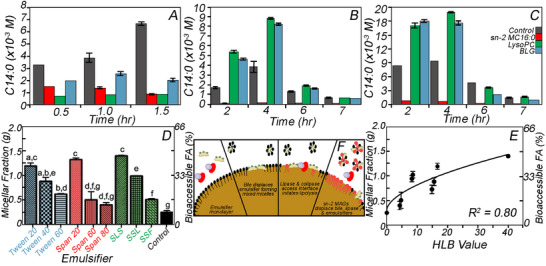
Tricaprylin‐in‐water emulsions, with sn‐2 monopalmitin (sn‐2 MC16), lysophosphatidylcholine (LysoPC), β‐lactoglobulin (BLG), or without an emulsifier (control) in the jejunum (A), ileum (B), and combined TIM‐1 total bioaccessible fraction (C) [59]. The combined bioaccessible caprylic acid fraction of the jejunum and ileum (D), when emulsified with Tweens, Spans, and charged surfactants, and its correlation with the HLB value of emulsifiers (E), along with an illustration of the lipolysis stages at the surface of an oil droplet (F) [62]. Data adapted from Reis et al., Food Biophysics, DOI: 10.1007/s11483‐008‐9091‐6 and Ng et al., Food & Function, DOI: 10.1039/c9fo02210d.

The first TIM‐1 study of lipid digestion focused on monodispersed oil‐in‐water (O/W) emulsions of tricaprylin, emulsified with *sn‐2* monopalmitin (*sn*‐2 MC16), lysophosphatidylcholine (LysoPC), or β‐lactoglobulin (BLG), compared with a control without an emulsifier [59]. BLG is the only emulsifier hydrolyzed by digestive enzymes, while sn‐2 MC16 and LysoPC remain unaffected by digestive enzymes. The results showed that gastric lipolysis slowed when emulsifiers were present in the food matrix (Figure 5A); however, the inhibition of pancreatic lipolysis (i.e., in the duodenum, jejunum, and ileum) only occurred for the *sn‐2* MC16 (Figure 5B,C) [59]. The concentration of amphiphilic compounds in the gastric compartment is low; thus, an emulsifier can impede gastric lipolysis by restricting lipase access to the interface, as bile and colipase are required to displace the engineered interface. In the TIM‐1 jejunum (Figure 5B) and ileum (Figure 5C) compartments, emulsifying the oil with LysoPC or BLG increased lipolysis compared to the control, and only sn‐2 MC16 slowed lipolysis. BLG hydrolyzes into small peptides, thereby reducing its interfacial activity and facilitating lipase access to the oil droplet surface. LysoPC is more surface‐active than lipase and thus inhibits gastric, but not intestinal, lipolysis because LysoPC solubilizes into bile salt micelles. These results suggest that the emulsifier sn‐2 MC16 is more interfacially active and efficiently displaces lipase and triglyceride from the oil–water interface by forming an interfacial monolayer. The sn‐2 MC16 monolayer is lipase‐resistant, as the enzyme is regiospecific for the sn‐1 and sn‐3 esterified fatty acids. Due to its high interfacial activity, the sn‐2 MC16 dominates the interface throughout digestion [60]. Reis et al. showed that, during intestinal digestion, adding an emulsifier other than sn‐2 MC16 to jejunal or ileal filtrates significantly enhances lipolysis and FFA release [59]. The systematic decrease in TAG hydrolysis for emulsions stabilized with sn‐2 MC16 occurred due to the exclusion of lipase and TAGs from the water–oil interface, leading to the saturation of the solubilization capacity of bile [59]. The ability to modulate lipolysis using emulsifiers represents a straightforward approach to enhancing the health outcomes of next‐generation ultra‐processed foods. Instead of designing emulsified foods that primarily focus on product stability, accounting for their potential to modulate bioaccessibility makes nutritional aspects central to future design.

In another TIM‐1 study, also using tricaprylin‐in‐water emulsions, nine emulsifiers selected based on comparable aspects of their low‐molecular‐mass structures were digested (Figure 5D) [61]. The three types of emulsifiers included Spans (Span 20 (sorbitan monolaurate), 60 (sorbitan monostearate), and 80 (sorbitan monooleate)) that differ only in hydrocarbon length, Tweens or polysorbates produced through ethoxylation of sorbitan differ based on the number of polyethylene repeats with 20, 60, and 80 tested; and ionic charged emulsifiers (sodium lauryl sulphate (SLS), sodium stearoyl lactylate (SSL) and sodium stearyl fumarate (SSF)). All surfactants significantly increased the total bioaccessible FFA in the combined jejunum and ileum (as reported in the previous study, the sum of Figure 4B,C), except for Span 60 and 80, which showed non‐significant increases (Figure 5D) [59, 61]. The control without emulsifier (Figure 5D) had the lowest bioaccessible FAs [61], supporting the previous conclusion that sn‐2 MAG accumulation at the interface limits lipolysis by displacing lipase from the oil interface when other surface‐active molecules are absent [59, 60]. Oils emulsified with Tween 20, Span 20, and SSL exhibited up to ∼4 times the lipolysis compared to the control. In contrast, other emulsifiers (e.g., Span 60 and 80) did not show more significant lipolysis. To further compare the effects of emulsifier on FA bioaccessibility, emulsifier HLB (hydrophilic–lipophilic balance) values versus the total bioaccessible FA were compared; higher values correspond to emulsifiers that are more effective at removing or displacing sn‐2 MAGs, a product of enzymatic lipolysis, from the interface, allowing lipase continued access to the TAGs at the surface (Figure 5E) [61]. Clearly, the choice of emulsifier affects product quality and shelf life, and it also influences lipid digestion dynamics (Figure 5F). After the structured emulsion interface (1) enters the duodenum, the pH is neutralized, and bile, digestive enzymes, and colipase mix with the emulsions. Thereafter, the emulsifiers displace from the interface and co‐solubilize into mixed bile salt micelles (2), which allows lipase and colipase access to the oil droplet surface and the TAGs contained therein (3). If insufficient emulsifier remains after lipolysis (4), sn‐2 MAGs displace lipase, limiting the extent of lipid digestion. A critical advantage of dynamic digestion models, including the TIM‐1, is their ability to remove lipid digestion products that otherwise accumulate and limit lipolysis.

### 1.5 Lipid Crystallinity and Acid Stability of Oil‐in‐Water Emulsions

Physicochemical properties differ between dietary triacylglycerols (TAGs); TAGs high in saturated FAs tend to be solid at room temperature, compared to unsaturated TAGs, which remain liquid due to their lower solid fat content (SFC) [38]. Yet lipid physicochemical properties, such as TAG crystallinity (including polymorphism) and the colloidal fat crystal network structure, as well as emulsification (including interfacial properties), can alter the bioavailability of fat‐soluble bioactives. The impact of lipid physical chemistry on postprandial lipemia, satiety response, and the bioavailability of lipophilic bioactives is understudied and often overlooked entirely, as TAG composition typically differs between these systems [63, 64, 65]. Crystalline TAGs are more challenging to digest compared to liquid TAGs; for example, in vitro, the digestibility of undercooled tripalmitin O/W emulsions hydrolyzed faster and more extensively than the crystallized emulsion [66]. An inverse relationship exists between SFC and in vitro lipid digestibility, as demonstrated by a human clinical trial comparing postprandial plasma TAG concentrations for crystalline solid versus liquid undercooled palm stearin (PS) emulsion droplets in healthy male participants [67].

**FIGURE 6:**
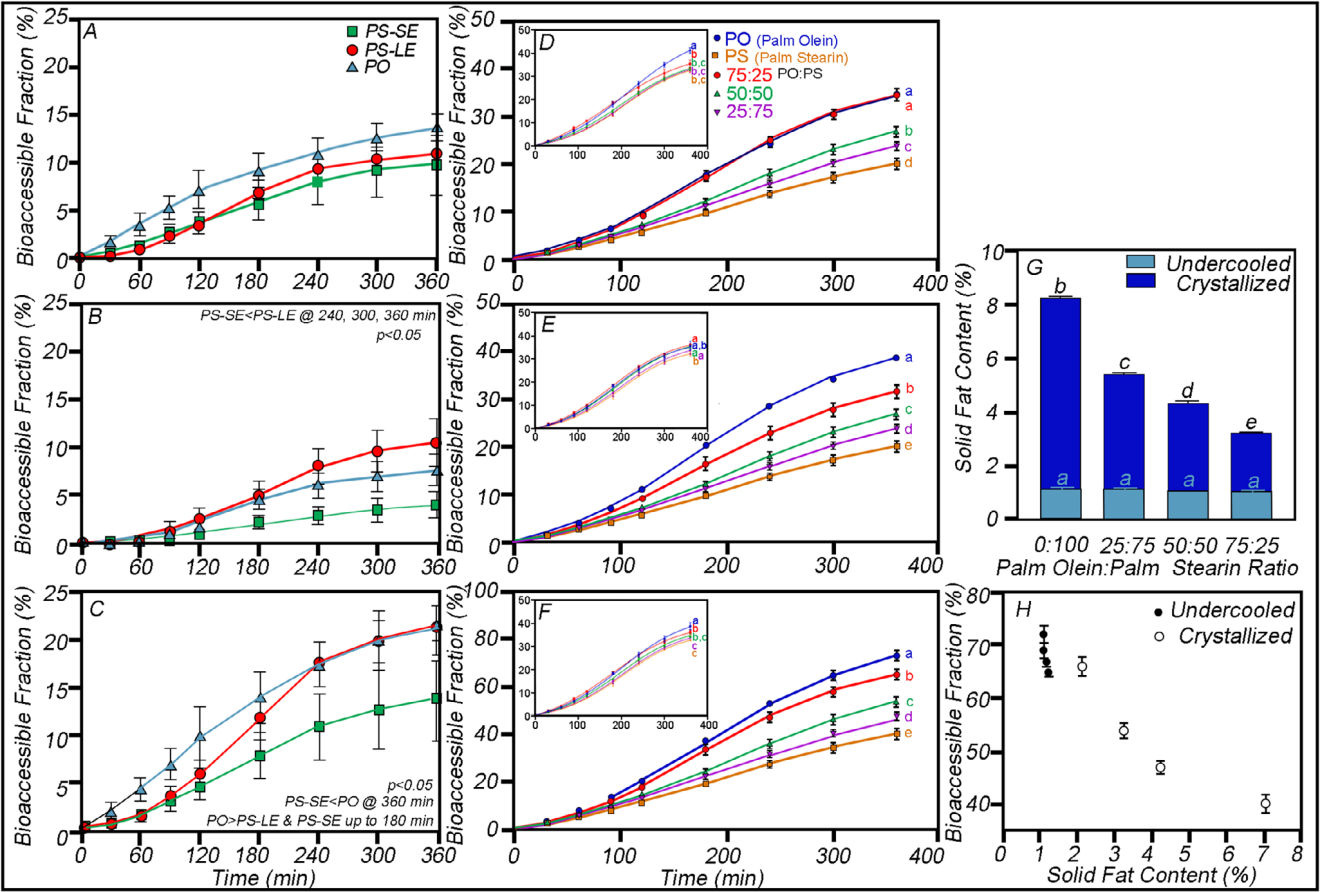
Percent bioaccessible FA fraction (A–C) in the jejunum (A), ileum (B), and combined TIM‐1 compartments during 6 h in vitro digestion of PS‐SE, PS‐LE, and PO [68]. Data reported as mean ± SEM, *n* = 4, with statistical differences determined by one‐way ANOVA. Cumulative FA bioaccessible fractions (D–F) obtained from the jejunum (D), ileum (E), and combined (F)) TIM‐1 compartments for crystallized (CR) (E,F) and undercooled (UC) (insets) emulsions of palm olein and palm stearin at incremental ratios (0:100; 25:75, 50:50; 75:25, and 100:0)) [69]. One‐way ANOVA with Tukey's multiple comparison tests revealed significant differences (*p* < 0.05) in the final FA bioaccessible fraction at 360 min. Solid fat content by pNMR for UC and CR O/W emulsions at 37°C (G), the correlation between the final bioaccessible FA fraction (H). Error bars represent mean ± 95% confidence interval ranges. Data from Li et al., Food & Function, https://doi.org/10.1039/D2FO03782C & Borduas et al., Food Chemistry, https://doi.org/10.1016/j.foodchem.2022.132326.

Hot homogenization of either palm olein (PO) or palm stearin (PS) O/W emulsions (10 w/v%) stabilized with 0.4 w/v% Span 60 followed by tempering obtained: (1) a control liquid state (PO), (2) an undercooled PS emulsion (PS‐LE) (at 37°C), and (3) a PS solid emulsion (PS‐SE) (crystallized in an ice‐water bath before storing at 4°C for 24 h (Figure 6A–C)) [68]. The PS‐SE, PS‐LE, and PO emulsions were monodisperse (peak diameter of ∼420 nm) and not significantly different based on volume‐weighted means (D_4,3_) for PO (0.5 ± 0.0 µm), PS‐LE (0.5 ± 0.0 µm), and PS‐SE (0.5 ±0.0 µm) (*p* > 0.05). Span 60 imparted strong electrostatic repulsion with statistically similar (*p* > 0.05) ζ‐potential values (e.g., PO ∼−44.0 ± 1.0, PS‐LE ∼−44.0 ± 1.0, and PS‐SE ∼‐37.2 ± 4.2 mV, respectively) [68]. PS‐SE, PS‐LE, and PO O/W emulsions were digested in the TIM‐1 system, and the bioaccessible FA fractions obtained over time for the jejunum (Figure 6A), ileum (Figure 6B), and combined (Figure 6C) compartments. Although the jejunal bioaccessibility trended higher for PO than for PS‐SE (Figure 6A), the difference was not significant (*p* > 0.05). Ileal bioaccessibility differed based on both time and treatment (*p* < 0.05), with no time x treatment interaction (*p* > 0.05) (Figure 6B) [68]. The FA bioaccessibility in the ileal filtrate of the TIM‐1 indicated that PS‐LE (8.0 ± 3.6%) was significantly greater than PS‐SE (2.8 ± 1.9%) after 240 min. The cumulative FA bioaccessibility of the combined jejunal and ileal compartments showed a significant effect of treatment (*p* < 0.05), where PO FA bioaccessibility was higher for the first 180 min than PS‐LE and PS‐SE (*p* > 0.05), which are similar to each other. After simulated digestion, at 360 min, the bioaccessible FA for PS‐SE (13.8 ± 9.0%) was lower than PO (21.6 ± 3.8%, *p* < 0.05), but was not significantly different from PS‐LE (21.3 ± 7.2%) (*p* = 0.06). As well, PS‐LE does not differ from PO (*p* > 0.05) (Figure 6C). The authors concluded that the degree of crystallinity in similarly sized o/w emulsions stabilized with Span 60 attenuated the FA release during early digestion [68].

In a study with palm‐based lipids, 10% O/W emulsions at PO: PS ratios from 0:100, 25:75, 50:50, 75:25, to 100:0 stabilized with 0.8 wt% Tween were hot homogenized and the emulsions were either equilibrated at 4^°^C in an ice water bath and stored at 4^°^C overnight to ensure maximum crystallinity (CR) or placed in a 37^°^C incubator, preventing nucleation, ensuring an undercooled liquid emulsion (UC) [69]. No flocculation or coalescence was observed at any PO/PS ratio, either crystallized or undercooled. As well, the droplet size distributions remained constant and monomodal across all emulsions (i.e., UC (223.8 ± 12.3 nm) and CR (235.0 ± 20.6 nm)) over 1 week due to the strong electrostatic repulsion with zeta potential values between −27.7 ± 2.6 mV for the CR and −31.3 ± 4.9 mV for the UC emulsions. The primary advantage of the experimental design was that each PO: PS ratio emulsion was compositionally identical, and either crystalline (Figure 6D–H) or undercooled liquids (Figure 6D–H insets), which are digested in the highly reproducible environment of the TIM‐1 to obtain FA bioaccessibility in the jejunum (Figure 6D), ileum (Figure 6E) and combined (Figure 6F). For the undercooled emulsions, FA bioaccessibility varied between 62% and 72%, with increasing PS levels lowering FA bioaccessibility. In contrast, in the CR emulsions, the FA bioaccessibility ranged from 40% to 65%, with statistically significant differences between PO:PS ratios. The SFC of UC was consistently low and not significantly different (between ∼1%‐–1.5%). In comparison, the CR emulsions decreased significantly with an increasing proportion of PO, that is, from 8% solids in PS to 4% in 75:25 PO:PS (Figure 6G). From this, the total bioaccessible FA versus SFC shows that chemical composition (UC emulsions) is less significant in determining FA bioaccessibility than the presence of solid fat (Figure 6H) [69]. This finding presents an exciting opportunity to develop next‐generation ultra‐processed foods that are digested in a manner designed for optimal nutrition, beyond composition.

**FIGURE 7:**
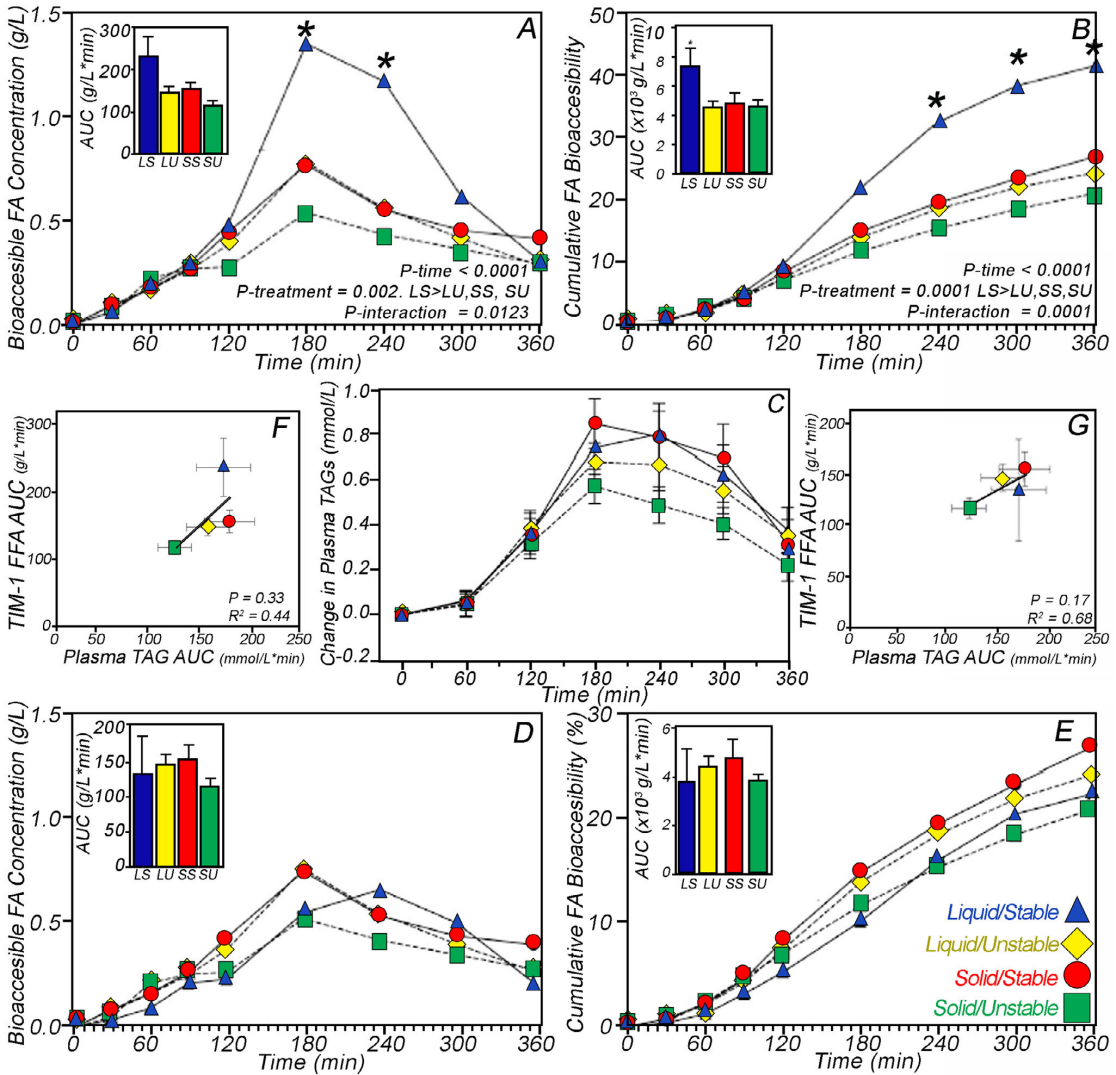
TIM‐1 bioaccessible FAs (A,D) and cumulative FA bioaccessibility (%) (B,E) (inset bar graphs show AUC of LS, LU, SS, and SU) using the standard TIM‐1 protocol (A,B) and modified gastric emptying rate (D,E) (*n* = 3). Data reported as mean ± SEM (asterisk indicates a significant difference between LS and all other emulsions (*p* < 0.05)) [71]. Postprandial changes in plasma TAG concentration in healthy men consuming 250 mL of SS, SU, LS, and LU (C) [70] and the correlation with the FA bioaccessibility from the standard (F) and modified gastric empty rate (G) TIM‐1 protocols [70].

The emulsion structure, exposure to mucin, and gastric pH can destabilize the emulsion, leading to flocculation, phase separation, and delayed gastric emptying. Our most significant and complex use of the TIM‐1, compared in vitro and in vivo results from a clinical trial (clinicaltrials.gov registration NCT03990246) for acid‐stable and acid‐unstable emulsions, which are either undercooled or crystalline (Figure 7) [70]. Fifteen healthy adult males on four occasions consumed 250 mL of either 20% palm stearin or palm olein emulsions containing partially crystalline droplets that remained stable (SS) or destabilized (SU) when exposed to simulated gastric conditions or liquid droplets that remained stable (LS) or destabilized (LU) under simulated gastric conditions [24]. Acid‐stable colloids in the liquid state, irrespective of TAG crystallinity, delayed gastric emptying and enhanced satiety after consumption [70]. The clinical trial found no significant differences in postprandial plasma TAG (Figure 7C); however, the gastric antrum cross‐sectional area (which scales with stomach volume) decreased faster (*p* ≤ 0.01) after participants consumed the acid‐unstable emulsions (SU and LU) [70]. In vivo, the gastric emptying half‐times were significantly higher for the acid‐stable emulsions (LS ∼300 and SS ∼262 min vs. LU∼184 and SU∼190 min). Lipid structure and physical state also altered short‐term satiety ratings and the polypeptide Y (PPY) response, with participants having 65% and 59% lower 3‐h incremental area under the curve (iAUC) values, which excludes the area below fasting levels, for hunger (*p* = 0.021) and desire to eat (*p* = 0.031) of LU compared to the SU emulsions. The LS emulsion had higher 6 h iAUC values for ghrelin (141%; *p* = 0.023) and PYY (150%; *p* = 0.043) compared to SU treatment and higher glucagon‐like peptide‐1 (GLP‐1) compared to SU (38%; p = 0.016 and LU (76%, *p* = 0.001). Thus, the emulsions that flocculated in the stomach (SU and LU) led to faster emptying and lower short‐term appetite suppression [36].

Coinciding with the clinical trial, the same acid‐stable and ‐unstable emulsions were fed to the TIM‐1, and significantly higher FA bioaccessibility after 120 min was observed for LS compared with all other emulsions, LU (*p* = 0.005), SS (*p* = 0.023), and SU (*p* = 0.0003) (Figure 7A,B). The FA bioaccessibility of the TIM‐1 AUC values did not concur with human postprandial TAG AUC, leading to a poor correlation (Figure 7F) between the AUCs for the non‐cumulative FA bioaccessibility (Figure 7A) versus the AUC from the clinical trial (Figure 7C). This lack of correlation was hypothesized to arise because of the altered gastric emptying rates observed by ultrasound in the study participants. The initial TIM‐1 standard protocol (Figure 7A,B) uses an 80‐min gastric emptying half‐time for all emulsions; however, the in vivo gastric emptying half‐times for LS and SU are 223 and 150 min, respectively. Therefore, the TIM‐1 experiments were repeated (Figure 7D,E) for LS with the gastric emptying half‐time adjusted to the ratio (1.55) of the in vivo gastric emptying times observed for LS: SU, which equated to an emptying half‐time of 124 min. Increasing the gastric emptying rate in the TIM‐1 accordingly led to the conclusion that there were no significant differences between formulations, consistent with the clinical trial findings (Figure 7C). Notably, modifying the gastric emptying time significantly improved the correlation (Figure 7G) between the TIM‐1 AUC bioaccessible FA (Figure 7D) and the change in plasma TAGs (Figure 7C). They also highlight an important limitation of the TIM‐1: the inability to account for hormonal feedback mechanisms and altered gastric emptying rates. All of this emphasizes the critical role food structure, including changes in response to the gastrointestinal environment, can play in influencing metabolic response.

### 1.6 Chemical and Enzymatic Interesterification of Lipids

The *trans* fats ban in formulated foods due to associated adverse health effects creates a high demand for alternative hardstock fats, yet they are in short supply. One strategy to tailor lipid physical properties without incorporating *trans* fatty acids is to blend a hardstock fat, often a fully hydrogenated oilseed or vegetable oil (high in stearic acid), with a liquid oil (high in oleic acid). Interesterification of the lipid blend broadens the melting transition, emulating the spreadable profile of butter and other plastic fats. Naturally occurring TAGs tend to have restricted random FA distribution, placing saturated FAs (SFA) preferentially at sn‐1 and sn‐3. Monounsaturated (MUFAs) and polyunsaturated (PUFAs) are typically more prevalent in the sn‐2 position [72, 73]. Interestification cleaves ester bonds, removing FAs from a glycerol backbone. These FAs then rearrange positions on different glycerol molecules without altering the overall FA composition, thereby altering functional and nutritional properties [72]. Sn‐2 FAs are more efficiently absorbed because pancreatic lipase releases them as 2‐MAGs, which are preferentially absorbed by intestinal cells, thereby preventing insoluble soaps from forming during digestion.

**FIGURE 8:**
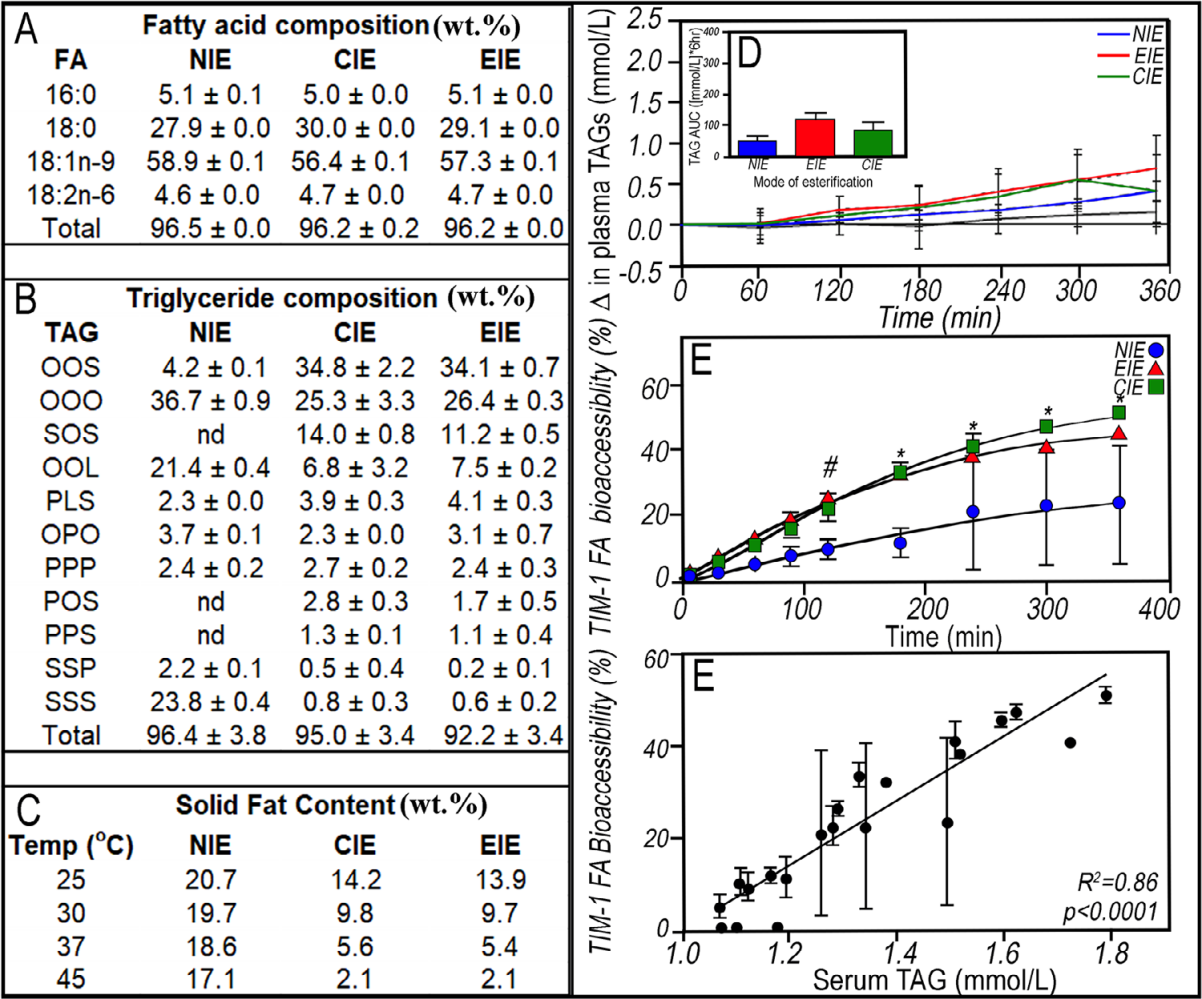
Fatty acid (A), triacylglycerol (B) composition, and solid fat content (C) of non‐interesterified (NIE), chemically interesterified (CIE), and enzymatically interesterified (CIE) 70:30 wt% blend of high‐oleic sunflower oil and fully hydrogenated canola oil [74]. Postprandial plasma TAG concentrations from baseline after ingestion of NIE (blue circle), EIE (red triangle), and CIE (green square) with bread in non‐obese healthy participants (*n* = 10) (D) [74]. TIM‐1 FA Bioaccessibility (%) of NIE, CIE, and EIE over 6‐h *i*n vitro digestion (*n* = 2) (E), [75] with data reported as mean ± SEM (# = Significant (*p* < 0.05) treatment difference between NIE and EIE; * = Significant (p < 0.05) treatment differences between NIE and both IE blends; overall ANOVA *p* = 0.013 for time × test fat interaction). Correlation analyses (F) between the human study (D) and TIM‐1 bioaccessibility (E) fed the same test fats (bars indicate the SEM, *n* = 2). Data from Robinson et al. Lipids. doi: 10.1007/s11745‐008‐3253‐7, and Thilakarathna et al., Food & Function, DOI: 10.1039/c5fo01272d.

In one study, 70 wt% high‐oleic sunflower oil (76% oleic acid) was combined with 30 wt% fully hydrogenated canola oil (88% stearic acid), heated to 80°C to ensure thorough mixing, and subjected to either chemical (CIE) or enzymatic (EIE) interesterification [74]. After determining the physicochemical properties of the non‐interesterified lipid blend (NIE) and the chemically (CIE) and enzymatically (EIE) interesterified blends, the lipids were digested by TIM‐1 and consumed by human participants to compare digestibility [75]. CIE lacks selectivity and cannot control the FA distribution; instead, FAs undergo random redistribution on TAGs until thermodynamic equilibrium among all possible TAG combinations [72]. Enzymatic interesterification using lipases directs the exchange of FAs at sn‐1 and sn‐3, allowing for exchange only between those positions; however, under certain conditions, EIE alters the acyl group at the sn‐2 position, which has applications in structured lipids in emulating human milk fat, improving fat absorption from infant formulas (IF) [76] In this case, CIE used a sodium methoxide catalyst for 60 min at 85^°^C, followed by quenching with citric acid. In contrast, EIE used a non‐specific Candida antarctica lipase immobilized on polyacrylic resin for 24 h at 70°C [74]. Interesterification does not alter the FA profile (Figure 8A); instead, it randomizes the FA position on glycerol TAG backbones, resulting in a broader range of TAGs. Postprandial lipidemia [77] after consuming the interesterified fat blends, determined in a randomized crossover study with 10 non‐obese and 11 obese males [74], was compared with lipid bioaccessibility in the TIM‐1 [75]. Inclusion of non‐obese and obese participants as postprandial plasma TAG concentrations differ significantly based on their different cardiometabolic risks. The NIE blend contained 21.7% OOL, which decreased to 3.6% for CIE and 7.7% for EIE; while OOS increased from 4.1% in the NIE blend to 36.9% and 33.4% in the CIE and EIE blends, respectively (Figure 8B). Altering the TAG composition alters the SFC of the lipid blends (Figure 8C). The NIE blend had the highest SFCs at 25°C (20.7%) and 45°C (17.1%) due to the presence of fully saturated TAGs present in canola stearin. Following interesterification, SFC decreases compared to NIE and is more sensitive to temperature. For example, CIE has 14.2% SFC at 25°C, which decreases to 2.1% SFC at 45°C, while EIE is 13.9% SFC at 25°C and 2.1 % SFC at 45°C [54].

Postprandially, the concentrations of serum stearic and oleic acid FAs increased following consumption of the CIE and EIE, while only oleic acid increased with NIE [74]. NIE contains significant amounts of tristearin, which does not melt until ∼70°C, making it less bioaccessible than the CIE and EIE lipid blends, where stearic acid is distributed across more TAG species, each with relatively lower melting temperatures than SSS [74]. The subset of non‐obese participants showed no significant differences in responses to NIE, CIE, and EIE, suggesting effective lipid metabolism and minimal disturbance of blood lipid homeostasis and plasma TAG accumulation (Figure 8D) [54]. In obese individuals, CIE resulted in significantly higher plasma TAG levels over 6 h compared to NIE. At the same time, NIE and CIE were not substantially different from EIE (data not shown) [74]. TIM‐1 FA bioaccessibility was determined for the NIE, CIE, and EIE lipids based on the cumulative FA bioaccessibility from the combined jejunal and ileal filtrates over a 6 h digestion protocol (Figure 8E). In contrast to the clinical trial, the physical state of NIE affected TIM‐1 digestion dynamics after 240 min; both interesterified lipid blends had significantly higher bioaccessibility than NIE. The TIM‐1 FA bioaccessibility and the postprandial TAG concentration in healthy nonobese participants showed a significant positive correlation (R2 = 0.8640; *p* < 0.05) (Figure 8F) [75]. In healthy participants, no significant differences in postprandial serum TAG appear; however, there was a trend toward higher TAG with CIE and EIE compared with the non‐interesterified blend (Figure 8D). This finding is consistent with the TIM‐1 results, which show lower bioaccessibility for the non‐interesterified blend compared with the interesterified lipid blends (Figure 8E) [67]. The TIM‐1 is focused on lipolysis and potential bioavailability based on events occurring in the gastrointestinal lumen, whereas blood lipid analysis reflects a broader range of metabolic processes. Thus, combining in vitro and in vivo approaches provides a more complete picture of the role of physical properties in metabolism.

### 1.7 Infant Formula and Human Breastmilk

Infant formula (IF) was first commercialized in 1867, before which the only alternatives were wet nursing (breastmilk (BM) from someone other than the mother) and dry nursing (e.g., milk from other mammals, predigested foods, and wheat‐containing porridges) [78]. Since then, several technological developments have occurred, although chemical analyses of dairy and human milk fat (HMF) highlight significant differences in TAG composition, protein and carbohydrate levels, and types [79]. Compared to IF, BM is more optimal for early‐life nutrition, as it is more energetically dense, promoting leaner growth while reducing the risk of obesity and metabolic disease in later life [79]. Nuturis, an IF developed by Danone, contains relatively large lipid droplets (3‐5 µm) coated with milk phospholipids and is more similar to BM than traditional IFs, which typically have particle sizes in the sub‐micron range [80]. Human BM particle size increases from 2.8 ± 0.3 to 8.9 ± 1.0 µm during lactation, with an average size of 4.0 µm during late stage lactation [81]. While a specific mechanism for the beneficial health outcomes associated with BM is not fully understood, it is postulated that different lipid droplet characteristics impact their gastrointestinal digestion, where sequential physical and chemical processes occur (e.g., gastric phase separation and emptying rate, lipolysis kinetics and the enteroendocrine cholecystokinin response, which alters gastric motility and secretion of bile) [79, 80]. The FA in BM (∼ 3%–5% total weight) are 98% esterified as TAGs, which supply almost 50% of the infant's calories (with the remaining provided by lactose and protein), and lipids are essential in facilitating the absorption of fat‐soluble vitamins (A, D, E, K) [78]. Two percent of the lipids are polar in nature and located within the milk fat globular membrane (MFGM). These include phospholipids and sphingolipids (<1%), free sterols and sterol esters (∼0.5%), small amounts of 2‐MAGs and DAGs and nonesterified free fatty acids [82].

Since IF commercialization in ∼1915, there has been a focus on humanizing the composition to emulate BM with a combination of vegetable oils to match BM's FA composition. Subsequently, in 1935, the protein concentration decreased, and finally, in 1962, the whey: casein ratio was altered to match that of human milk [78, 83]. These adaptations aimed to mimic the BM protein, carbohydrate, and fat ratio, leading to Similac (“similar to lactation”) IF [78]. Part of the challenge in mimicking human milk fat (HMF) is the differences in FA composition between colostrum, transitional, and mature milk [84]. After matching the FA profile of BM, the sn‐position FA are unique in HMF, where palmitic acid is preferentially located at sn‐2 (∼70% of C16:0 at sn‐2), contrary to most vegetable oils, where an unsaturated FA exists at sn‐2. Sn‐2 palmitic acid prevents this saturated FA from being hydrolyzed, after which it could form an insoluble soap with calcium and be excreted, thereby reducing both lipid and calcium bioavailability [85]. Instead, palmitic acid at sn‐2 is preferentially absorbed as an sn‐2 MAG. FA composition changes between colostrum, transitional, and mature milk, corresponding to increased medium‐chain fatty acid (MCFA) (∼10%–15%) [84]. Unique to HMF, the majority of TAGs contain one MCFA and two LCFA (MLL‐TAG). In contrast, vegetable oils contain a greater proportion of TAGs with three MCFAs (MMM‐TAG), and other mammalian milks contain two MCFAs and one LCFA (MML type) [86].

**FIGURE 9:**
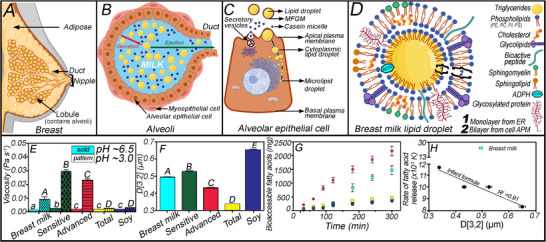
Illustration of lactation (A–C) and the formation of the complex milk fat globular membrane (D). Viscosity at pH 3.0 and 6.5 (E) and the droplet surface moment mean diameter D[3, 2] (F) for Sensitive, Total Comfort, Advance, and Soy Similac IFs compared to human BM (letters represent significant differences from one‐way ANOVA (*p*<0.05) and a Tukey's Multiple Comparison Test). The cumulative FA TIM‐1 bioaccessibility from the combined jejunum and ileum (G) correlated with the Sauter mean diameter of each IF (H). Data from Fondaco et al., Food Biophysics. DOI 10.1007/s11483‐014‐9388‐6 [88].

The breast comprises two structures related to lactation: lobules, which grow and develop during pregnancy, and ducts (Figure 9A). Lactogenesis, the process by which alveolar cells undergo maturation and gain the ability to secrete or eject milk into ducts by contraction of myoepithelial cells (Figure 9B), is initiated after delivery and removal of the placenta, caused by hormonal changes that follow. Mammary or alveolar epithelial cells biosynthesize and secrete proteins, lipids, and lactose in milk (Figure 8C). Lipids are synthesized at the endoplasmic reticulum surface and form microdroplets that coalesce in the cell, becoming cytoplasmic lipid droplets. Cytoplasmic lipid droplets reach the apical plasma membrane and are enveloped by the membrane during secretion, leading to the formation of the MFGM that stabilizes the O/W emulsion. The MFGM is a tri‐layer membrane with a complex composition (e.g., phospholipids, sphingolipids, gangliosides, choline, sialic acid, cholesterol, and membrane proteins) (Figure 9D), comprised of the initial monolayer formed by the endoplasmic reticulum organelle membrane. The bilayer is compositionally similar to the apical plasma membrane. At the same time, the Golgi apparatus assembles amino acids into whey and casein proteins, where they are phosphorylated and chelated with calcium before being transported and released by secretory vesicles at the apical plasma membrane [87]. Due to the secretory process, an extremely complex O/W interface exists in BM, but not in IF. It is interesting to note that a significant component of the MFGM is phospholipids, which increase lipase access to the TAGs at the droplet surface [59, 60].

Improvements in IF have focused on minimizing physical property differences with BM. In one study, the initial pH, viscosity (Figure 9E), and particle size distributions (Figure 9F), as well as the viscosity at gastric pH (∼3.0 for infants) (Figure 9E), were compared between BM and four Similac IFs (Sensitive, Total Comfort, Advanced, and Soy) [88]. The initial pH ranged from 6.5 to 7 before adding HCl to reduce it to 3.0, simulating the gastric environment. At pH 6.5, BM viscosity is significantly lower than IFs (Figure 9F, lower case). At gastric pH (3.0), the viscosity increased significantly for both IF and BM. The BM viscosity at pH 3.0 was statistically greater (*p* >0.05) than Total Comfort and Soy, but lower than Sensitive and Advanced (Figure 9E). The particle size distribution for BM, at pH 6.5, showed three size distributions (∼0.1, 1.0, and 7.0 µm), whereas Total Comfort was monomodal (0.4 µm). The remaining formulas present two distinct peaks at 0.4 and 3.0 µm [88]. The particle size distributions (PSD), converted to the Sauter or volume‐weighted mean [D3,^2^], indicate that all systems have significantly different particle sizes, with Total Comfort being the smallest, followed by Advanced and Sensitive. At the same time, the largest D [3, 2] is for Soy IF. In contrast, BM's D [2, 3] is significantly greater than the Advanced and Total Comfort formulas but lower than the Sensitive and Soy formulas (Figure 9F). Thus, significant differences exist between IFs and BM in D [3, 2] and viscosity, suggesting that lipid bioaccessibility differs based on these attributes.

To probe FA bioaccessibility, each sample standardized to 5 g of fat was fed to the TIM‐1 system. The total FA bioaccessibility at 300 min of the combined jejunal and ileal filtrates (Figure 9G), from highest to lowest was Advance (∼74.5 ± 6.1 %), BM (∼50.6 ± 5.3 %), Total Comfort (∼24.5 ± 5.6 %), Sensitive (∼14.1 ± 3.9 %), and Soy (∼12.4 ± 2.7 %). Fatty acid bioaccessibility was significantly (*p* < 0.05) higher for Advance than for BM, and both were higher than for Total Comfort, Sensitive, and Soy formulas. Total Comfort, Sensitive, and Soy formulas do not show statistically significant differences. After fitting the total bioaccessible FAs from the jejunal and ileal compartments combined to the three‐parameter shifted logistic model, the maximum bioaccessibility (%), the rate of FA release (mg/min), and the critical time, which is the time where half of the total amount of FA is released, were determined. The fitted maximum bioaccessibility shows similar trends (Advanced > BM > Total Comfort > Soy > SensitiveTM) to the bioaccessible FA at 300 min simulated digestion from the in vitro digestion. The critical time encompasses the initial lag period, the FA release rate, and the duration of lipolysis. The time taken to release half of the bioaccessible FAs was shorter for all formulas compared to BM, starting at the slowest to most rapid: Soy (38 min), followed by Total Comfort (41 min), Sensitive (48 min), Advanced (79 min), and BM (175 min). The extended critical time required for BM may arise from an initial inhibitory effect of the native milk fat globular membrane and the time needed for bile to displace other interfacially adsorbed molecules, allowing lipase to access the oil–water interface. The final shifted‐logistical model parameter, the rate constant, indicates that the kinetics of FA release occur within a narrow range for the IF (0.008–0.012 mg/min); however, the rate constant of BM is double that of the IFs (0.025 mg/min). This finding highlights the challenges of formulating colloidal IF that yield digestion dynamics similar to those of BM.

The Tiny‐TIM is a specialized in vitro model that has an advanced gastric compartment simulating the fundus and antrum, but only a single intestinal compartment. A study with the Tiny‐TIM showed that the size and coating of IF and BM alter lipid handling (e.g., lipid droplet coalescence, flocculation, phase separation) and bioaccessibility [79]. The droplet characteristics altered phase separation during gastric digestion, where the large phospholipid‐coated droplets present in BM and Nuturis migrated to the top layer more rapidly than other IF, due to their larger particle sizes, and were retained in the gastric compartment longer than IF [74] Smaller lipid droplets in IF remained homogenous for the first 120 min, after which IF without MFGM forms larger gastric aggregates, as MFGM limits lipid droplet aggregation but not the creaming rate [79]. Gastric emptying is predetermined to follow a standardized profile in the Tiny‐TIM and does not differ between IF and BM; however, phase separation alters the rate of gastric emptying for the lipids, specifically, where lipids in IF empty faster and more steadily than in BM or IF with MFGM. BM follows a slower, biphasic lipid emptying rate, resulting in altered lipolysis kinetics and lower lipid bioavailability [74]. These findings also concur with those described above for phospholipid emulsifiers [59, 60], emulsion acid stability [70], and FA bioaccessibility of Similac IFS [88]. This example illustrates how studies utilizing the TIM models facilitate the examination of food structural changes within the gastrointestinal tract.

### 1.8 Lipids Embedded in Whole Foods—Eggs, Steak, and Burgers

**FIGURE 10:**
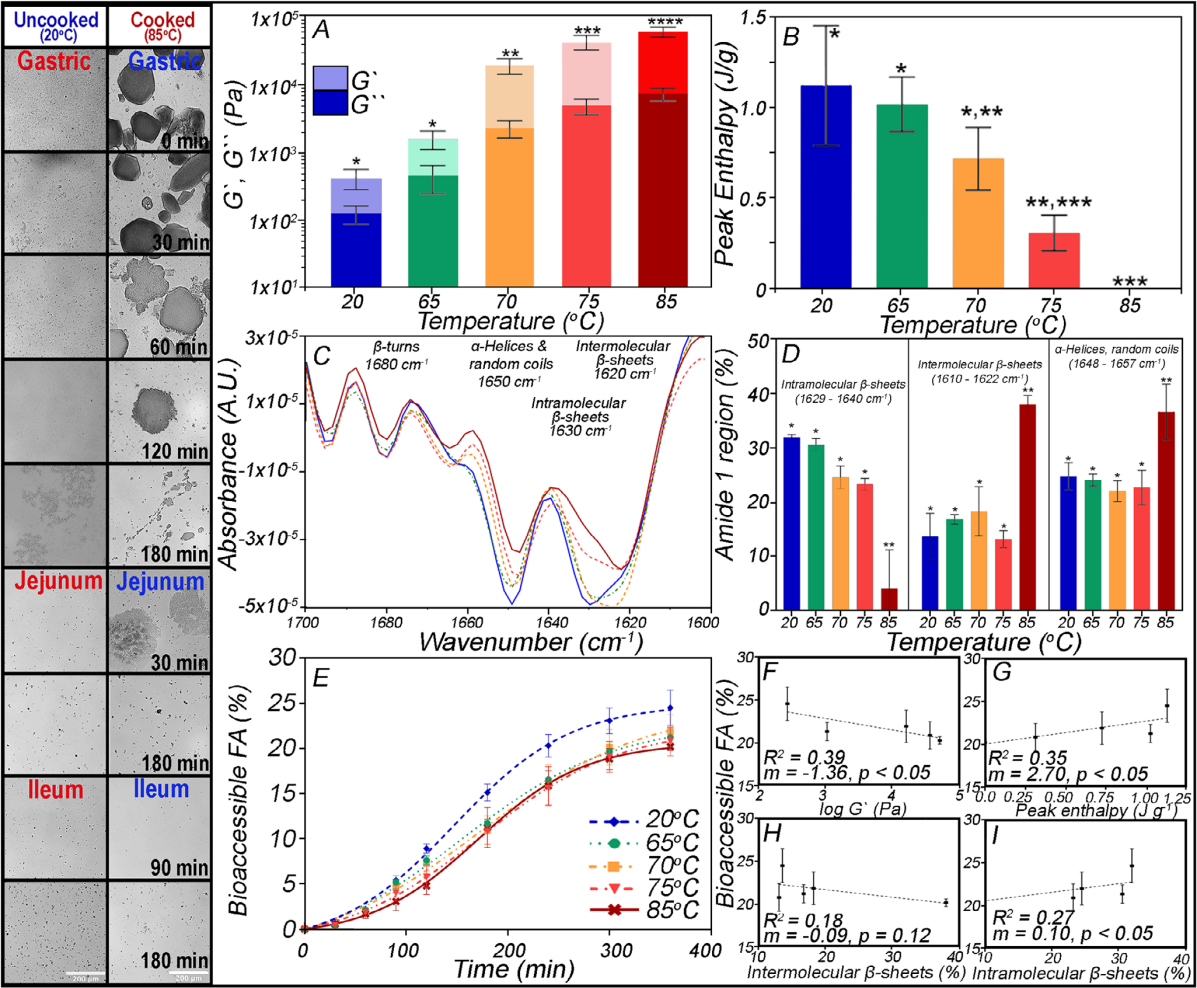
Effect of temperature on the physical properties (*G*` and *G*`` (A), peak denaturation enthalpy (B), FTIR absorbance of the amide one region (C), and the ternary configuration (D) obtained from FTIR). Micrographs illustrate changes in the microstructure of raw and cooked egg yolk digesta in the TIM‐1 Gastric, jejunum, and ileum compartments and the cumulative bioaccessible FAs from the jejunum and ileum TIM‐1 compartment combined (E). The total bioaccessible FA from the TIM‐1 correlates inversely to *G*` (F) and the quantity of intermolecular β‐sheets (H) and linearly correlates to peak enthalpy (G) and intramolecular β‐sheets (I). Data from Xu. Et al, Food Biophysics, DOI:10.1007/s11483‐021‐09699‐3 [94].

The food microstructure alters the bioaccessibility and bioavailability of macro‐ and micronutrients [5, 23, 89]. For example, the extent of processing almonds drastically affects lipid digestion and caloric density [90, 91, 92, 93]. To maintain an identical lipid composition but investigate the role of food microstructure in lipid digestion, eggs‐in‐shell each had a thermocouple inserted into their centre and were placed in a water bath at 65^°^C, 70^°^C, 75^°^C, or 85^°^C, until they equilibrated to the set temperature. After cooking, the physiochemical properties of separated egg yolks were assessed, including viscoelastic (Figure 10A) and thermal (Figure 10B) properties as well as changes in protein secondary structure using Fourier‐transform infrared spectroscopy (FTIR) of the amide I region (1600–1700 cm^−1^) before (20°C) and after (65^°^C, 70^°^C, 75^°^C, 85°C) cooking (Figure 10C) [94]. The egg yolk viscosity increased when cooked at 65°C compared to the raw egg yolk; however, the egg yolk did not coagulate, forming a solid‐like gel until >70°C. Thermal egg protein denaturation caused coagulation and increased the rheological elastic or storage modulus (G`). Tanδ is the ratio of the *G*″ to *G*′ to quantify the relative energy dissipation, where a higher Tanδ value indicates a greater tendency for the material to dissipate energy, suggesting a more viscous behavior. In comparison, a lower value indicates a more elastic behavior. Tan δ was less than 0.2 for eggs cooked above 70^°^C, forming a firm gel, while at 65^°^C, and for the uncooked egg, tan δ∼0.5 remained liquid (Figure 10A). The increase in elasticity corresponds to a decrease in the normalized transition enthalpy (Figure 10b) obtained from differential scanning calorimetry. The reduced transition enthalpy after cooking suggests a gradual loss of native protein structure with increasing temperature. Comparing the denaturation enthalpy of each cooked egg to that of the uncooked (raw) egg provides an approximate extent of denaturation that corresponds to a decrease in observed enthalpy. Using the amide I region of FTIR, a loss in native protein structure was apparent through the depletion of intramolecular beta‐sheets and an increase in intermolecular beta‐sheets and random coils (Figure 10C,D).

When the egg yolks were digested in the TIM‐1 system (Figure 10E), there was a steady increase in bioaccessible FAs up to 180 min. Afterward, bioaccessibility steadily declined, leading to differences in the final bioaccessible FA. Raw yolks and those cooked to 65°C had higher bioaccessible FA (18%–25%) than those cooked to 85°C (14%). At the same time, the intermediate cooking temperatures did not significantly differ in FA bioaccessibility [94]. Significantly (*p* < 0.05) non‐zero slopes suggest FA bioaccessibility is inversely proportional to log *G*` (Figure 10F), proportional to transition enthalpy (Figure 10G), and intramolecular β‐sheets (Figure 10I), and does not correlate to intermolecular β‐sheets (Figure 10H). Thus, as the network elasticity (G`) increases, due to protein denaturation (enthalpy decreases, and loss of intramolecular β‐sheets), FA bioaccessibility declines as it slows the disintegration of the bolus and remaining food microstructure in the luminal digestive fluids, delaying lipase access to the emulsified TAGs, and slowing lipolysis. Micrographs of raw and 85°C yolks show much larger, denser aggregates in the cooked egg than in raw. Before digestion, particle size analysis of the micrographs revealed that 75% of the particles in raw eggs were smaller than 10 µm. In comparison, the cooked yolk had only 15% due to lipid–protein aggregation and the formation of aggregates exceeding 1000 µm [94]. Through digestion, the fraction of particles < 30 µm in the cooked yolk increased from 50% at *T* = 0 min in the gastric phase to 90% at 360 min in the ileal compartment; similarly, the largest particles (>100 µm) decreased from 30 to 10 % of the distribution in the first 120 min of digestion. Since FA bioaccessibility is greater in raw yolks, it would suggest that small particles regulate lipid digestion more than the rate of protein matrix degradation found in 85^°^C yolks.

**FIGURE 11:**
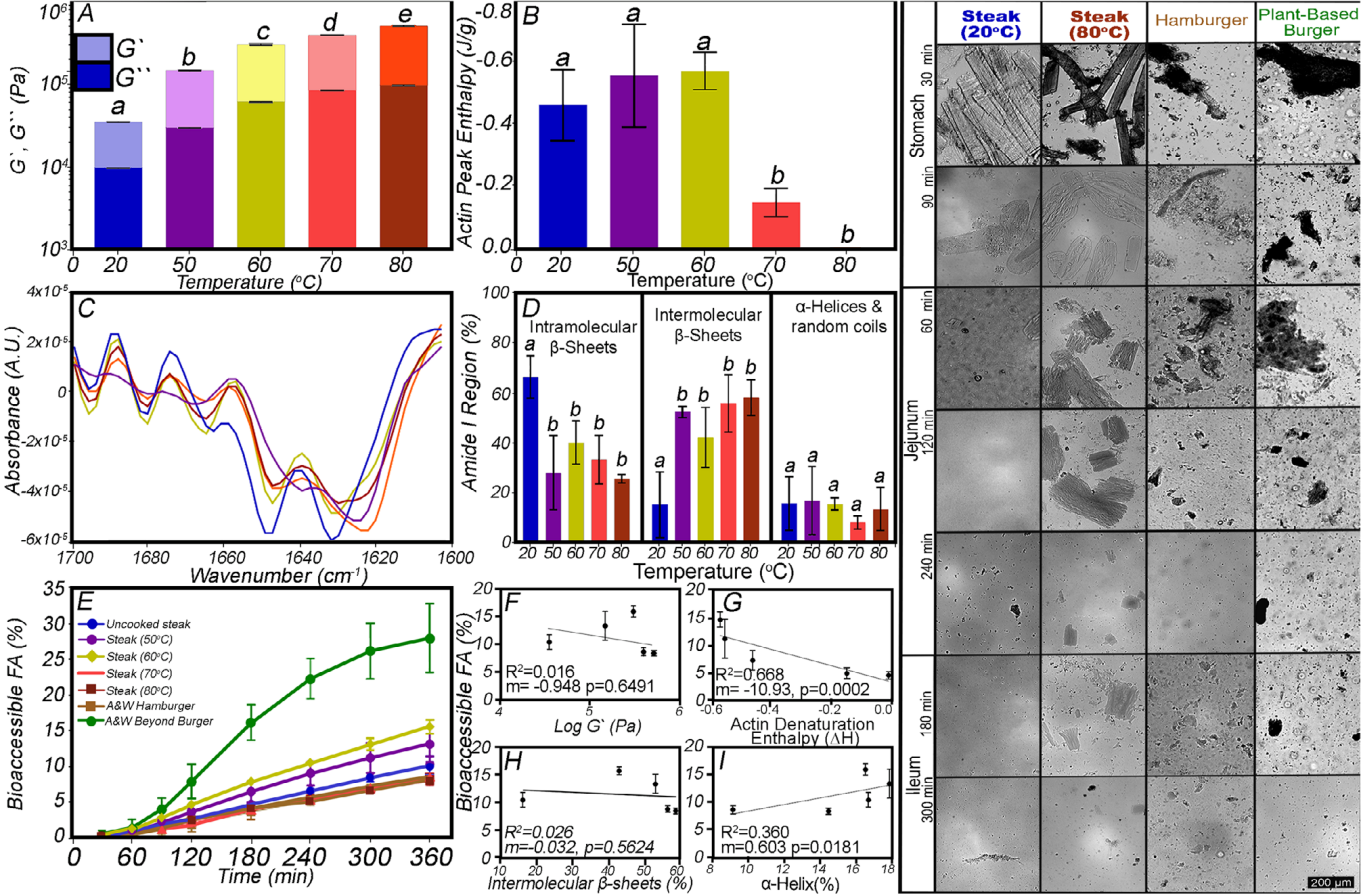
Effect of sous vide temperature on the physical properties of steak (*G*` and *G*`` (A), peak denaturation enthalpy (B), FTIR absorbance of the amide one region (C), and the ternary configuration (D) obtained from the amide 1 FTIR region). Micrographs illustrate changes in the microstructure of raw and cooked steak digesta in the TIM‐1 Gastric, jejunum, and ileum compartments and the cumulative bioaccessible FAs from the jejunum and ileum TIM‐1 compartment combined (E).

Similar experiments investigated the effect of the food matrix on lipid bioaccessibility from beef samples. These were 2.5 cm‐thick cuts from Canadian AAA boneless beef striploin (Longissimus lumborum) (6 days postmortem) with subcutaneous fat removed before undergoing a similar cooking protocol to the egg yolks. The striploin was vacuum‐sealed before sous vide cooking in a preheated water bath at 20°C (raw), 50°C, 60°C, 70°C, and 80°C, and removed when the internal core temperature equilibrated to the set water temperature [32]. Proximate analysis was conducted to determine the macronutrient composition before (11.6% fat, 64.6% moisture, and 22.1% protein) and after cooking (4.6% fat, 69.3% moisture, and 23.6% protein) to ensure that TIM‐1 meal sizes were based on the same concentration of TAGs. Similar to the egg yolks, the effect of cooking temperature on the physicochemical properties of steak was investigated using *G*`/*G*`` (Figure 11A), denaturation enthalpy (Figure 11B), and protein secondary structure (Figure 11D) obtained from the amide I region of the FTIR spectra (Figure 11C). Cooking induced similar physicochemical changes between the egg yolk and steak, as the temperature positively associates with *G*`, and inversely with peak enthalpy. Cooking also induced a red shift in the amide I region of the FTIR spectrum, as aggregated proteins exhibit increased transition dipole coupling due to enhanced hydrogen bonding in the intermolecular β‐sheets [95].

As the intramolecular β‐sheets declined, intermolecular β‐sheets increased, while no change in random coils or α‐helices was present. These findings suggest that the food matrix alters upon protein denaturation, changing how macronutrients are embedded within and limiting their exposure to luminal fluid and digestive enzymes, ultimately affecting the FA bioaccessibility (Figure 11E). The primary difference observed from the egg yolk is that the raw steak was solid, while the uncooked egg and the egg cooked to 65°C were liquid. As such, one would expect less significant effects of the food matrix on FA bioaccessibility. At 360 min of digestion, the maximum FFA bioaccessibility increased (*p* < 0.001) to 60°C; cooking temperatures above 60°C significantly decreased FFA bioaccessibility [32]. The only physical parameter to correlate to FA bioaccessibility was the protein denaturation enthalpy of actin at 75.6 ± 0.48°C (*p* = 0.0083, one‐tailed Spearman's rank correlation coefficient *ρ* = −1.0) (Figure 11G). No other parameters correlated (log *G*` (*p* = −0.60, one‐tailed Spearman's rank correlation coefficient *ρ* = 0.1750) (Figure 11F), intermolecular β‐sheets (Figure 11H) (*p* = −0.70, one‐tailed Spearman's rank correlation coefficient *ρ* = 0.1167) and α‐helices (Figure 11I) (*p* = 0.60, one‐tailed Spearman's rank correlation coefficient *ρ* = 0.1750)) [32] Actin is a key structural element of the thin filament in the sarcomere. When denatured, it coincides with meat toughening, making it harder to digest [32]. Micrographs of raw meat digestion show rapidly degrading fiber structure in the gastric phase (up to 90 min), with only microscopic fiber remnants emptying into the jejunum. Meat cooked to 85°C exhibited the highest actin denaturation and elastic modulus, making fibers more challenging to erode and digest, resulting in more significant fiber remnants remaining throughout the 360‐min simulated digestion.

In a subsequent study, plant‐based Beyond burgers and hamburgers purchased from A&W were also digested in the TIM‐1 to compare lipid bioaccessibility [96]. The cumulative FA bioaccessibility was plotted against the degree of doneness (DOD) for boneless beef striploin (Figure 11E). FA bioaccessibility of the hamburger did not significantly differ from the 85°C DOD striploin. The comminuted meat in hamburger reduces the particle size compared to the striploin; however, the high cooking temperature (cooked on a double‐sided grill at 165°C for 2.5 min) causes actin to denature, expelling water, and creating more dense aggregates that persist through digestion, similar to the 85°C DOD striploin. From the TIM‐1 cumulative combined jejunal and ileal bioaccessible fractions and micrographs, it is evident that plant‐based burgers do not digest similarly to meat, as FA bioaccessibility is three‐fold larger than that of meat, irrespective of whether it is comminuted or the DOD, as plant‐based burgers lack the whole food structure that embeds lipids, restricting them from reaching the luminal fluid and digestive enzymes. In these studies of meat, TIM‐1 enabled a better understanding of how protein cooking alters microstructure in the gastrointestinal tract, thereby affecting lipid digestion. The results offer insights into potential differences between plant‐based and animal meats that have relevance to health.

## 2 Conclusions

The concept of food structure is highly relevant in various contexts. In designing the next generation of foods, structure must remain at the forefront of product development when considering palatability, including for ultra‐processed products. However, efforts must expand to include consideration of how the microstructure alters nutrient release throughout digestion and, ultimately, bioaccessibility, much as in a pharmaceutical delivery system. Designing healthful foods in the future will require knowledge of the structure‐function relationships of food ingredients, their interactions, the resultant food microstructures, and macroscale physical properties throughout formulation, processing, storage, as well as the biochemical and biophysical aspects of digestion. In particular, as our fundamental understanding of how food structure affects glycemia and lipidemia expands, nuanced formulation changes will enable the design of foods that tailor these responses to support metabolic health, potentially incorporating the concept of the lipemic index. Tools such as the TIM‐1 are yielding critical insights to support these efforts. All of this presents the exciting opportunity to reconsider aspects of food formulation to enhance nutritional outcomes beyond a focus on food composition and the utility of in vitro digestion models.

## Funding

The authors have nothing to report.

## Participant Consent

No identifiable information was included; thus, no written consent has been obtained from the participants.
